# Effect of Vitamin D Receptor Activation on the AGE/RAGE System and Myeloperoxidase in Chronic Kidney Disease Patients

**DOI:** 10.1155/2017/2801324

**Published:** 2017-12-06

**Authors:** Claudia Torino, Patrizia Pizzini, Sebastiano Cutrupi, Rocco Tripepi, Antonio Vilasi, Giovanni Tripepi, Francesca Mallamaci, Carmine Zoccali

**Affiliations:** ^1^CNR-IFC, Clinical Epidemiology and Physiopathology of Renal Diseases and Hypertension, Reggio Calabria, Italy; ^2^Nephrology and Renal Transplantation Unit, Reggio Calabria, Italy

## Abstract

Vitamin D receptor (VDR) activation has been reported to increase circulating levels of the advanced glycation end products (AGE) and their decoy receptor (RAGE). However, until now, the effect of VDR activation on AGE and RAGE has not been tested in the setting of a randomized, double-blind clinical trial. We have therefore analyzed the effect of VDR activation by paricalcitol on pentosidine, S100A12/ENRAGE, and RAGE and on established biomarkers of oxidative stress like myeloperoxidase in CKD patients in the PENNY trial. At baseline, human S100A12/ENRAGE, RAGE, and myeloperoxidase, but not pentosidine, were intercorrelated, and the association between S100A12/ENRAGE and myeloperoxidase (*r* = 0.71, *P* < 0.001) was the strongest among these correlations. Paricalcitol failed to modify biomarkers of the AGE/RAGE system and myeloperoxidase in unadjusted and adjusted analyses by the generalized linear model (GLM). No effect modification by other risk factors was registered. Paricalcitol does not modify biomarkers of the AGE/RAGE system and myeloperoxidase in CKD patients. The apparent increase in RAGE levels by VDR activation reported in previous uncontrolled studies is most likely due to confounding factors rather than to VDR activation per se. This trial is registered with NCT01680198.

## 1. Introduction

The vitamin D receptor (VDR) is part of the superfamily of nuclear receptors that regulate several genes containing a vitamin D-responsive gene promoter element. VDR-responsive genes are involved in cell proliferation and differentiation, membrane transport, cell adhesion, matrix mineralization, inflammation, and oxidative stress [1]. Mitigation of oxidative stress is considered a major pathway implicated in the renal and cardiovascular protective effects of VDR activation [2]. Several biological mechanisms may lead to oxidative stress, and multiple biomarkers of oxidative stress exist [3]. Among these mechanisms, stimulation of the advanced glycation end product (AGE) receptor by a low-molecular weight AGEs like pentosidine or by compounds of the S100/calgranulin family like S100A12/ENRAGE is a relevant pathway leading to cardiovascular disease and renal damage in CKD patients [4]. On the other hand, the circulating receptor of AGE (RAGE) acts as a decoy receptor and affords protection from cardiovascular disease in the same patients [5, 6]. As to myeloperoxidase, oxidants derived from the activity of this enzyme such as hypochlorous acid may critically interfere with several cell functions thereby engendering tissue and organ damage. Myeloperoxidase gene ablation [7] prevents renal injury after surgical removal of the 4/5 of renal mass in the rat, and high myeloperoxidase levels are considered relevant for the progression of renal disease and cardiovascular complications in the CKD population [8]. We have previously shown that pentosidine, a major AGE, is a marker of concentric remodeling in dialysis patients [9] and that circulating soluble RAGE correlates inversely with atherosclerosis [5] and left ventricular hypertrophy [6] in patients with chronic kidney disease (CKD). Furthermore, in a secondary analysis in the paricalcitol and endothelial function in chronic kidney disease (PENNY) trial [10], we have recently observed that pentosidine modifies the sclerostin response to VDR activation by paricalcitol [11]. However, until now, there is no randomized clinical trial that tested the effect of VDR activation on AGE and RAGE and on myeloperoxidase in CKD population. With this background in mind, we have now made a thorough analysis of the effect of paricalcitol treatment on pentosidine, S100A12/ENRAGE, and RAGE circulating levels in the PENNY trial.

## 2. Materials and Methods

The study protocol was approved by the ethics committee of our institution. A written informed consent was obtained from each participant.

### 2.1. Patients

The protocol of the PENNY trial and the corresponding CONSORT flow diagram are detailed in the previous paper describing the main results of the study [10]. Briefly, the PENNY trial is a double-blind, randomized, parallel-group trial (ClinicalTrials.gov identifier: NCT01680198) which enrolled 88 patients with CKD stages 3 to 4. The inclusion criteria were age ranging between 18 and 80 years, parathormone ≥ 65 pg/mL, serum total Ca between 2.2 and 2.5 mmol/L, and phosphate levels between 2.9 mg/dL and 4.5 mg/dL. The exclusion criteria were treatment with vitamin D compounds or antiepileptic drugs and the presence of neoplasia, symptomatic cardiovascular disease, or liver disease. Patients who met the inclusion criteria were randomized (1 : 1) to receive 2 *μ*g paricalcitol once daily or matching placebo for 12 weeks after a 2-week run-in. Measurement of relevant variables in the PENNY trial was made at baseline, after 12 weeks of treatment with paricalcitol or placebo, and again 2 weeks after stopping these treatments. The dose of paricalcitol was adjusted on the basis of serum parathormone and Ca, and the maximum dose allowed was 2 *μ*g daily. No vitamin D compounds were allowed during the trial. Demographic, clinical, and biochemical data of the two study arms are listed in Table 1.

### 2.2. Laboratory Measurements

Serum calcium, phosphate, glucose, and lipids were measured in the routine clinical pathology laboratory at our institution. Serum creatinine was measured by the Roche enzymatic, IDMS-calibrated method and serum cystatin C by the Siemens Dade Behring kit, and the GFR was calculated by the CKD-Epi creatinine-cystatin formula [12]. Plasma parathormone was measured by an immunoradiometric assay (DiaSorin, Stillwater, MN, USA) and 25-OH VD and 1,25-OH VD by a radioimmunoassay (Immunodiagnostic Systems, Boldon, UK). Serum human RAGE, myeloperoxidase, S100A12/ENRAGE, and plasma pentosidine were measured by validated ELISA methods by using commercially available kits by R&D Systems (Minneapolis, MN) (human RAGE and myeloperoxidase), MBL International (Woburn, MA) (S100A12/ENRAGE), and Cusabio (College Park, MD) (pentosidine). The intra- and interassay coefficients of variation (CV) for each kit are the following: human RAGE: 5.7%–7.7%; myeloperoxidase: 2.1%–9.0%; S100A12/ENRAGE: 4.3%–5.4%; and pentosidine: <8%–<10%. Serum and plasma samples were kept frozen at −80° degrees, without freeze-thaw cycles, until analysis, and biomarker measurements were performed in a single assay.

### 2.3. Statistical Analysis

Data are reported as mean ± standard deviation (normally distributed data), median and interquartile range (nonnormally distributed data), or percent frequency, and comparisons between groups were made by independent *t*-test, Mann–Whitney test, or chi-square test. Correlates of markers of oxidative stress (human RAGE, S100A12/ENRAGE, myeloperoxidase, and pentosidine) were analyzed by using Pearson's correlation coefficient (on log_10_-transformed data, when appropriate) and linear regression analyses. The effect of paricalcitol on these biomarkers after 12 weeks of treatment was analyzed by applying the generalized linear model (GLM). Differences in risk factors at baseline not controlled by randomization and due to chance were accounted for by introducing the same risk factors in the GLM. The effect sizes of paricalcitol on the outcome measures in this study were summarized by the generalized eta squared (*η*^2^), as recommended by Bakeman [13]. The changes in biomarkers of oxidative stress in paricalcitol-treated and untreated patients after stopping the interventions (paricalcitol and placebo) were investigated by using the paired *t*-test applied to the measurements made at the 12th week (end of the trial) and to those made 2 weeks after the end of the trial. The potential effect modification by demographic (age and gender) and bone mineral disorder biomarkers at baseline (calcium, phosphate, 25-OH vitamin D, 1,25-OH vitamin D, PTH, and FGF23) on the relationship between allocation arm and markers of oxidative stress was investigated by standard interaction analyses by introducing into the models' appropriate multiplicative terms [14]. Data analysis was performed by SPSS for Windows (version 24.0, Chicago, Illinois, USA).

## 3. Results

At baseline, patients randomized to paricalcitol and placebo did not differ for demographic, clinical, and biochemical characteristics, except for the eGFR which tended to be higher (*P* = 0.06) in patients receiving paricalcitol and FGF-23 which tended to be lower (*P* = 0.07) in the same patients (Table 1). No patient had vitamin D deficiency (25-OH VD levels < 10 ng/mL), whereas vitamin D insufficiency (25-OH VD levels > 10 ng/mL to <30 ng/mL) was noticed in 26 patients in the PCT group and in 19 patients in the placebo group (*P* = 0.20). Alongside comparable plasma levels of bone disorder biomarkers—including serum calcium and phosphate, 25-OH vitamin D, 1,25-OH vitamin D, PTH, and FGF23—the average values of S100A12/ENRAGE, pentosidine, RAGE, and myeloperoxidase at baseline were very similar in the two study arms (Table 1). As detailed in the source study [10], drug treatments, including ACE inhibitors, sartans, hypoglycemic agents, statins, and proton pump inhibitors, were similar between the two groups except for calcium carbonate, more frequently administered in patients on placebo (22.7%) than in those on the paricalcitol arm (0%) (*P* = 0.003).

### 3.1. Intercorrelations of AGE/RAGE and Myeloperoxidase in CKD Patients and Other Functional Relationships of These Biomarkers

**Figure 1:**
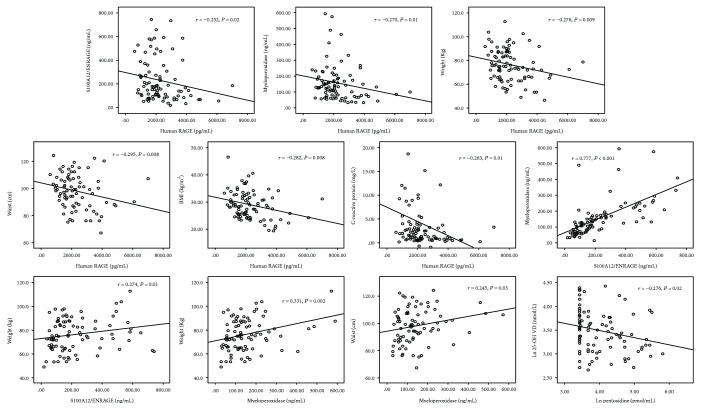
Main correlates of human RAGE, S100A12/ENRAGE, myeloperoxidase, and pentosidine.

At baseline, human S100A12/ENRAGE, RAGE, and myeloperoxidase, but not pentosidine, were mutually correlated (Table 2) and the correlation between S100A12/ENRAGE and myeloperoxidase was the strongest among these correlations (Figure 1).

S100A12/ENRAGE, RAGE, and myeloperoxidase coherently associated with body weight (S100A12/ENRAGE: *r* = 0.274, *P* = 0.01; human RAGE: *r* = −0.276, *P* = 0.009; and myeloperoxidase: *r* = 0.331, *P* = 0.002) while pentosidine did not (*r* = 0.176, *P* = 0.10). Human RAGE (*r* = −0.295, *P* = 0.008) and myeloperoxidase (*r* = 0.245, *P* = 0.03) correlated also with waist circumference while S100A12/ENRAGE (*r* = 0.202, *P* = 0.07) and pentosidine (*r* = 0.17, *P* = 0.30) did not. Finally, among these biomarkers, RAGE was the sole to correlate with C-reactive protein (*r* = −0.263, *P* = 0.01). Apart from the direct link between 25-OH vitamin D and pentosidine (*r* = 0.254, *P* = 0.02), no correlation was found between the same biomarkers and biomarkers of bone mineral disorder (PTH, 1,25-OH vitamin D, and FGF23).

### 3.2. Effect of Paricalcitol on Biomarkers of Oxidative Stress

**Figure 2:**
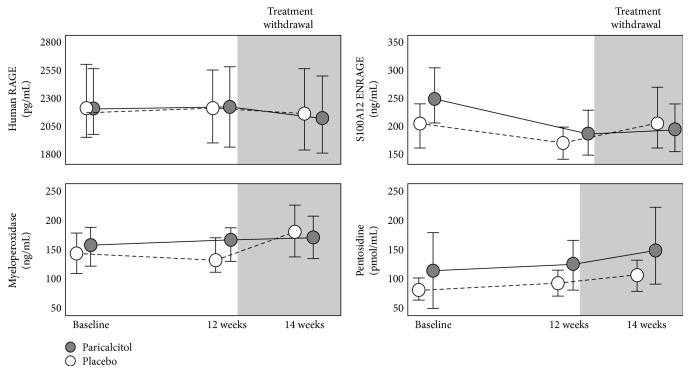
Effects of paricalcitol on human RAGE, S100A12/ENRAGE, myeloperoxidase, and pentosidine after 12 weeks of treatment and 2 weeks after stopping paricalcitol. Data are expressed as mean and 95% CI.

After a 12-week treatment, paricalcitol suppressed PTH and 1,25-OH_2_ vitamin D, producing a modest rise in serum calcium and phosphate, a marked rise in FGF23, and no change in 25-OH VD (see Supplementary Figure 1 and [10]). However, vitamin D receptor activation by this drug largely failed to modify S100A12/ENRAGE, pentosidine, RAGE, and myeloperoxidase (Figure 2 and Table 3). These results did not change after adjustment for the variables that differed at baseline between the study arms, that is, eGFR, calcium carbonate treatment, and FGF23 (Table 3). Diabetes did not modify the effect of paricalcitol treatment on AGE and RAGE (all *P* for effect modification ≥ 0.173). Effect modification analyses did not show any interaction with age, gender, baseline 25-OH vitamin D, 1,25-OH vitamin D, calcium, phosphate, PTH, and FGF23 (all *P* > 0.05). The levels of these biomarkers remained unchanged after stopping the treatment with paricalcitol/placebo (Figure 2).

## 4. Discussion

This study performed within the framework of the randomized clinical trial [10] shows that paricalcitol largely fails to modify biomarkers of the AGE/RAGE system and major biomarkers of oxidative stress like myeloperoxidase in CKD patients.

Protein glycation is a complex series of reactions occurring in all tissues and fluids where glucose reacts with proteins giving rise to a series of advanced glycation end products (AGE) [15]. Incomplete digestion of AGE-modified protein results in the formation of low-molecular weight degradation products incorporating AGE modifications including pentosidine, N(epsilon)-(carboxymethyl)lysine (CML), and free-imidazole AGEs [16]. Low-molecular weight (LMW) AGEs activate AGE-specific receptors (RAGE) while high-molecular weight AGEs do not activate this pathway and induce tissue and organ damage by a different mechanism [17]. LMW AGEs bind to RAGE in various tissues including the endothelium and tubule tissues in the kidney and induce vascular and renal damage via activation of the nuclear factor *κ*B, a major inflammatory pathway, and via the mitogen-activated protein kinase pathway [18]. Among LMW AGEs, pentosidine is seen as a powerful biomarker of AGE-dependent damage in disparate conditions including diabetes, aging, and CKD, particularly so in kidney failure [19]. Apart from pentosidine and LMW AGEs, the AGE receptor is also activated by S100A12/ENRAGE, an important ligand for this receptor that has been implicated in vascular inflammation, coronary and aortic atherosclerosis, and plaque vulnerability and in human cardiovascular disease [20].

In theory, stimulation of the VDR appears to be a relevant pathway whereby alterations in the AGE/RAGE pathway may be favorably affected in patients with CKD. Indeed, vitamin D supplementation mitigates the accumulation of AGEs in the vascular system in rats with streptozotocin-induced diabetes [21], and treatment with 1,25-OH vitamin D increased serum RAGE in an uncontrolled, sequential study in CKD patients on chronic dialysis [22]. Furthermore, in a nonrandomized study in vitamin-deficient women with ovary polycystic disease, treatment with 1,25-OH vitamin D increased RAGE levels, an effect that went along with a parallel decline in serum anti-Mullerian hormone levels, a critical alteration implicated in impaired folliculogenesis in these patients [23]. In a previous analysis in the PENNY trial, we observed that paricalcitol treatment, while not affecting circulating levels of pentosidine, modified the relationship between this AGE and sclerostin, a bone hormone which increases after treatment with both inactive vitamin D forms like cholecalciferol [24] and activated vitamin D compounds like paricalcitol [11]. With this background in mind, we set out to test the hypothesis that the AGE/RAGE system and myeloperoxidase levels in CKD patients may be favorably affected by treatment with paricalcitol. In this respect, the PENNY trial [10], a double-blind, randomized trial testing the effects of paricalcitol on CKD patients, offered the ideal setting for investigating this hypothesis. Indeed, serum samples for the measurement of the key biomarkers considered in the present study were available in all patients (no missing sample). Pentosidine, S100A12/ENRAGE, RAGE, and myeloperoxidase were measured by well-validated methods (see Materials and Methods) with very good intra- and interassay variability (<10%), and thorough analysis of the mutual correlations among these compounds showed consistent internal relationships suggesting that these biomarkers reflect AGE/RAGE status in CKD patients. Notably, myeloperoxidase was strongly associated with RAGE and AGE ligands, and RAGE associated with both body weight and waist circumference, a well-recognized metric of abdominal adiposity in CKD patients [25]. However, contrarily to our hypothesis, paricalcitol treatment failed to affect the circulating levels of RAGE ligands investigated in this study as well as myeloperoxidase levels. Notably, this was true both in unadjusted analyses by the generalized linear model as well as in analyses by the same model adjusted for variables that marginally differed in the study arms like the eGFR and treatment with calcium carbonate and FGF23.

Even though results in this study robustly negate that paricalcitol treatment may favorably affect the circulating levels of biomarkers of the AGE/RAGE system and of myeloperoxidase, circulating levels of these compounds may not adequately reflect levels of the same biomarkers at tissue level. Therefore, the fact that we did not measure the tissue levels of these biomarkers is a limitation of our study. Another limitation of this study is the small sample size, calculated on the primary outcome of the trial (i.e., modification in endothelial function) rather than on the (hypothetic) effect of paricalcitol on AGE-RAGE. Furthermore, all patients enrolled in this study were Caucasian and followed up in a single Nephrology Unit, and this might impair the generalizability of results. However, circulating levels of pentosidine [26], S100A12/ENRAGE [27], RAGE [5, 6], and myeloperoxidase [7] have already been associated with evidence of tissue damage and clinical events in CKD patients. Thus, failure of paricalcitol to modify these biomarkers implies that this drug is unlikely to meaningfully modify the potential risk for adverse clinical outcomes related to AGE accumulation in CKD. The strength of our study is that it is based on a randomized, double-blind trial with no missing blood sample throughout the trial.

## 5. Conclusions

In conclusion, paricalcitol does not modify biomarkers of the AGE/RAGE system and major biomarkers of oxidative stress like myeloperoxidase in CKD patients. Our data suggest that the apparent increase in RAGE levels during treatment with 1,25-OH vitamin D in previous studies in hemodialysis patients [22] and in women with polycystic ovary [23] is most likely due to uncontrolled confounding factors rather than to 1,25-OH vitamin D treatment.

## Figures and Tables

**Table 1:** Demographic, clinical, and biochemical characteristics of the two study arms at baseline.

	Active group (*n* = 44)	Placebo group (*n* = 44)	*P*
Age (years)	63 ± 11	62 ± 12	0.65
Male sex (%)	59%	70%	0.27
Current smokers (%)	12%	19%	0.37
Past smokers (%)	45%	41%	0.66
Diabetes (%)	34%	36%	0.82
BMI (kg/m^2^)	29 ± 5	29 ± 5	0.66
Systolic/diastolic BP (mmHg)	123 ± 16/73 ± 9	129 ± 21/73 ± 11	0.16/0.81
Heart rate (beats/min)	67 ± 8	68 ± 10	0.64
Cholesterol (mg/dL)	164 ± 41	162 ± 43	0.84
HDL cholesterol (mg/dL)	47 ± 11	50 ± 13	0.18
LDL cholesterol (mg/dL)	88 ± 34	88 ± 36	0.91
eGFR_Cyst_ (mL/min/1.73m^2^)	34 ± 12	29 ± 13	0.06
Hemoglobin (g/dL)	12 ± 2	12 ± 2	0.49
Calcium (mmol/L)	2.25 ± 0.12	2.21 ± 0.10	0.16
Phosphate (mmol/L)	1.20 ± 0.19	1.23 ± 0.16	0.29
Parathormone (pg/mL)	102 (81–146)	102 (85–154)	0.70
FGF-23 (pg/mL)	64.7 (52.7–81.2)	78.0 (53.7–103.1)	0.07
1,25-OH vitamin D (pmol/L)	101.4 ± 41.6	93.6 ± 41.8	0.32
25-OH vitamin D (nmol/L)	33 ± 16	38 ± 16	0.19
C-reactive protein (mg/L)	1.18 (0.68–3.02)	2.49 (0.99–3.74)	0.11
S100A12/ENRAGE (ng/mL)	165 (103–469)	175 (88–272)	0.39
Pentosidine (pmol/mL)	43.6 (31.2–108.9)	44.1 (31.2–99.5)	0.87
Human RAGE (pg/mL)	2072 (1571–2984)	2027 (1481–2794)	0.81
Myeloperoxidase (ng/mL)	128.5 (71.5–204.0)	127.8 (91.5–176.8)	0.90

Data are expressed as mean ± SD, median and interquartile range, or percent frequency as appropriate. BMI: body mass index; BP: blood pressure; LDL: low-density lipoprotein; HDL: high-density lipoprotein; eGFR: estimated glomerular filtration rate; FGF-23: fibroblast growth factor-23.

**Table 2:** Intercorrelations of biomarkers of oxidative stress.

	Human RAGE	S100A12/ENRAGE	Myeloperoxidase	Pentosidine
Human RAGE	1	*r* = −0.252, *P* = 0.02	*r* = −0.270, *P* = 0.01	*r* = −0.009, *P* = 0.93
S100A12/ENRAGE	*r* = −0.252, *P* = 0.02	1	*r* = 0.777, *P* < 0.001	*r* = −0.091, *P* = 0.40
Myeloperoxidase	*r* = −0.270, *P* = 0.01	*r* = 0.777, *P* < 0.001	1	*r* = −0.171, *P* = 0.11
Pentosidine	*r* = −0.009, *P* = 0.93	*r* = −0.091, *P* = 0.40	*r* = −0.171, *P* = 0.11	1

**Table 3:** Generalized linear models showing no effect of paricalcitol on serum human RAGE, myeloperoxidase, S100A12/ENRAGE, and plasma pentosidine after 12 weeks of treatment.

	Univariate	Adjusted for eGFR, calcium carbonate treatment, and FGF23
Human RAGE	*η* ^2^ = 0.000, *P* = 0.91	*η* ^2^ = 0.005, *P* = 0.54
S100A12/ENRAGE	*η* ^2^ = 0.005, *P* = 0.51	*η* ^2^ = 0.001, *P* = 0.83
Myeloperoxidase	*η* ^2^ = 0.016, *P* = 0.24	*η* ^2^ = 0.022, *P* = 0.18
Pentosidine	*η* ^2^ = 0.001, *P* = 0.74	*η* ^2^ = 0.005, *P* = 0.52

## Supplementary Materials

Supplementary materials Effects of Paricalcitol on biomarkers of mineral-bone disorder after 12 weeks of treatment and 2 weeks after stopping Paricalcitol. Data are expressed as mean and 95% CI.
