# Supplementary material for: Effect of Vitamin D Receptor Activation on the AGE/RAGE System and Myeloperoxidase in Chronic Kidney Disease Patients
Source: Oxid Med Cell Longev. 2017 Dec 6;2017:2801324. doi: 10.1155/2017/2801324 (PMC5737482; doi:10.1155/2017/2801324)
Supplement: Supplementary materials — Effects of Paricalcitol on biomarkers of mineral-bone disorder after 12 weeks of treatment and 2 weeks after stopping Paricalcitol. Data are expressed as mean and 95% CI. [file 2801324.f1.docx]

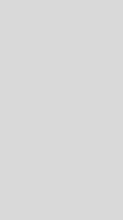

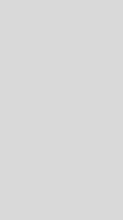

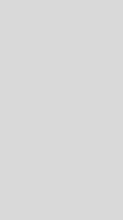

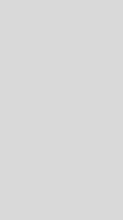

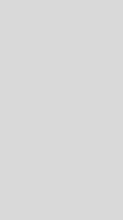

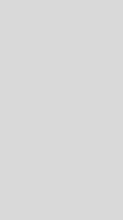


**PTH (pg/ml)**

220

180

140

100

60

**Ca (mMol/L)**

**P (mMol/L)**

**FGF23 (pg/ml)**

250

200

150

100

50

40

30

20

//

**1,25 OH_2_VD (pMol/L)**

50

40

30

//

**Baseline 12 weeks 14 weeks**

**25 OH_2_VD (nMol/L)**

**Baseline 12 weeks 14 weeks**

Treatment withdrawal

Treatment withdrawal

Placebo

Paricalcitol

3.0

2.5

2.0

1.6

1.4.

1.2

1.0

Suppl. Fig. 1
